# Validation of the Hedonic and Eudaimonic Motives for Activities-Revised Scale in Chinese Adults

**DOI:** 10.3390/ijerph18083959

**Published:** 2021-04-09

**Authors:** Wenjie Li, Linting Zhang, Ning Jia, Feng Kong

**Affiliations:** Department of Psychology, Shaanxi Normal University, 199 South Chang’an Road, Xi’an 710062, China; lwj10011001@163.com (W.L.); zhanglintingdebbie@126.com (L.Z.); energeticjenny@126.com (N.J.)

**Keywords:** hedonic and eudaimonic motives, confirmatory factor analysis, reliability, validity, measurement invariance

## Abstract

The Hedonic and Eudaimonic Motives for Activities-Revised scale (HEMA-R) is one of the most extensively used instruments to assess how people pursue well-being. The main aims of the present research were to translate HEMA-R into Chinese and test its construct and predictive validity as well as measurement invariance across gender. In Study 1, we conducted confirmatory factor analysis with data containing 1090 Chinese undergraduates, and replicated the two-factor model which has been found in other studies. Furthermore, the measurement invariance across gender was supported throughout the multi-group confirmatory factor analysis. Study 2 replicated these results and further found the HEMA-R had satisfactory predictive validity in measures of well-being, social support and smartphone addiction. All the findings indicate that the HEMA-R is reliable and valid to measure hedonic and eudaimonic motives, and it could be applied generally across gender in Chinese adults.

## 1. Introduction

With the flourishing of positive psychology, the way people pursue well-being has attracted numerous researchers to study. There are two types of pursuits: hedonia and eudaimonia [1]. These concepts have their roots in ancient philosophy. For instance, in the fourth century BCE, Aristippus pointed out that the object of life is enjoyment, no matter where it comes from. On the contrary, in the opinion of Aristotle, it is vulgar to seek only enjoyment. He thought pursuing well-being means searching for the deep purpose of life and developing the best in oneself [2]. To this day, the discussion between them still attracts a lot of researchers [3,4,5,6]. Hedonia sees people pursuing pleasure and enjoyment to acquire well-being, including physical, cognitive and emotional aspects. In other words, they seek enjoyment and comfort at this moment instead of in the future, whether it’s physically, intellectually or socially [5]. For eudaimonia, people try to actualize their potentials. The pursuit of such an objective leads them to apply and develop themselves to their fullest [7]. In the literature, hedonia and eudaimonia have been assessed as motives for activities [8,9,10,11,12]. Hedonic motives are defined as seeking pleasure and comfort, while eudaimonic motives refer to people seeking to try their best to use and develop themselves [9]. Through the years, researchers found that the eudaimonic and hedonic motives exhibited beneficial effects on a wide range of positive outcomes such as academic achievement, emotion regulation, coping strategies and well-being [12,13,14]. Thus, measuring and studying hedonic and eudaimonic motives is a vital and necessary topic.

To measure eudaimonic and hedonic motives, Huta and Ryan [9] have developed the Hedonic and Eudaimonic Motives for Activities (HEMA) scale. The scale contains nine items such as “Seeking to pursue excellence or a personal ideal?” (Eudaimonic motives) and “Seeking pleasure?” (Hedonic motives) [9]. In the research of Huta and Ryan [9], the results of confirmatory factor analyses (CFA) indicated that there existed a two-factor structure in this scale, and similar results have been found in some other studies [15,16]. Moreover, eudaimonic has often been defined in terms of meaning, Huta [17] added an item of “Seeking to contribute to others or the surrounding world?” and developed the revised version of the scale (i.e., the 10-item HEMA-R).

Over the years, the HEMA and its revised version have been translated into many other languages such as Croatian [15], German [18], Italian [18], Japanese [19], Persian [20], Polish [21], Romanian [22], and Swedish [18]. These studies manifested that the HEMA possessed great psychometric properties. However, the applicability of it to the Chinese population was still unknown, which was one of the intentions of the present study.

When using the HEMA, another critical question is the measurement invariance across different groups. Generally speaking, considering that gender difference is a basic trait for humans, gender invariance was the first to be examined. The difference between males and females in the HEMA scores has been explored by Huta [8], and Asano and Igarashi [19], and their findings manifested that there was no significant difference across gender in America and Japan. But Matranga and Restivo [23] found that the attitude to being hedonic was lower for females compared to males. Furthermore, when testing the gender difference, it is vital for us to make sure that the scale can measure the same construct across gender. However, to date, no study has investigated the measurement invariance of the HEMA across gender in China. Therefore, testing gender invariance of the HEMA in Chinese background was another objective of our research.

In addition, the predictive validity of the HEMA was also included in the research. The engine model of well-being is a framework for understanding the diversity of the entire field of well-being. According to this model, the input (i.e., external resources and internal characteristics) would influence the outcomes (i.e., actions characterized by well-being) through the processes (e.g., cognitive factors) [24]. Thus, the motives, as a cognitive factor in the processes, should influence well-being outcomes. Hedonic well-being reflects the hedonic aspects of well-being, while eudaimonic and social well-being reflect the eudaimonic or functioning aspects of well-being [25], so hedonic motives and eudaimonic motives may have different influences on them. Through the years, some researchers have shown that both hedonic and eudaimonic motives positively relate to hedonic well-being and eudaimonic well-being using the HEMA [9]. To be specific, hedonic motives tend to have stronger correlations with hedonic well-being than eudaimonic motives. In contrast, eudaimonic motives relate more closely to eudaimonic well-being than hedonic motives. [26]. However, the link between happiness motives and social well-being was still unknown. Thus, we would like to examine the relationship between them, and we assumed that the hedonic motives were linked to hedonic well-being significantly rather than social and eudaimonic well-being, while eudaimonic motives had the opposite results.

According to self-determination theory, individuals have an internal trend to grow and have inherent psychological needs, which form the basics of their motives, behaviors and psychological well-being [27]. These contain three kinds of immanent needs: autonomy, competence and relatedness. Relatedness mainly refers to being close and in contact with others, including families and friends. As one of the available social resources [28], social support could reflect the relationship between individuals and others. Thus, we supposed that the happiness motives may link to the social support from being close with others, besides, an empirical study has found this relationship [19]. Therefore, social support was chosen to test the predictive validity as well.

In addition, with the continuous progress of technology, smartphones have become a more and more prevalent media. According to the uses and gratifications approach (U&G), users select media stimuli on their own initiative so that their specific demands can be satisfied [29]. The U&G provided a view to account for the relationship between an individual’s pursuits and behaviors. Compared to those who pursue self-actualization, people who seek relaxation and enjoyment may choose media such as smartphones which could provide them with happiness and pleasure. Besides, some researchers have tested that hedonic motives were positively associated with internet addictive behavior [30]. Thus, we also assessed the predictive validity of smartphone addiction. Generally speaking, we expected that hedonic motives positively correlated with well-being, social support and smartphone addiction, and eudaimonic motives positively correlated with well-being and social support except for smartphone addiction.

In sum, we conducted two studies to address the construct and predictive validity as well as measurement invariance across gender of the HEMA-R in Chinese adults. In Study 1, we used a confirmatory factor analysis (CFA) to assess whether the two-factor structure existed in the HEMA-R in data from Chinese adults. Then, a multi-group CFA was performed to examine the measurement invariance across gender. In Study 2, we tried to explore whether the results were similar to those of Study 1 and examined the predictive validity of the Chinese version of HEMA-R in relation to well-being, social support and smartphone addiction.

## 2. Study 1

### 2.1. Methods

#### 2.1.1. Participants and Procedure

A total of 1090 emerging adults were recruited from one university in China, and the sample included 562 males and 528 females. Age information was not gathered, which is an important limitation of this study, but judging from the typical participant pool, we estimated the median age to be 20 years. In the sample, 452 were freshmen, 184 were sophomores, 212 were juniors and 242 were seniors. In the current research, each participant voluntarily joined, and signed written informed consent before this study. Then, they completed questionnaires on an online survey website (www.wjx.cn,). The study was approved by the institutional review board of the local university.

#### 2.1.2. Measurement

To measure hedonic and eudaimonic motives, we used the HEMA-R [17]. It is a self-reporting instrument consisting of two subscales, each contains five items. Sample items are as follows: “Seeking to pursue excellence or a personal ideal?” (Eudaimonic Motives) and “Seeking enjoyment?” (Hedonic Motives). Each item is answered with a 7-point Likert scale ranging from 1 (not at all), to 7 (very much). In this study, we translated the HEMA-R into Chinese using a standard back-translation procedure. Specifically, this scale was first translated into Chinese by two bilingual psychologists, and then another two bilingual psychologists who didn’t know the scale were invited to translate it back to Chinese. Then, the original experts checked this back-translated version of the HEMA-R. Finally, after regulating and revising minor words, the original psychologists confirmed the content validity of the back-translated version. In study 1, the Cronbach’s alpha coefficients of the hedonic motives and the eudaimonic motives were 0.903 and 0.901, respectively.

#### 2.1.3. Data Analysis

At first, to assess whether the initial two-factor structure existed in HEMA-R using a Chinese sample, we conducted a CFA using Mplus 7.4. The following indices were performed to test the applicability of models: comparative fit index (CFI), non-normal fit index (NNFI), root mean square error of approximation (RMSEA), and standardized root mean square residual (SRMR) [31]. Furthermore, the model would fit well if RMSEA and SRMR < 0.08, NNFI and CFI > 0.90.

Then, a multi-group CFA was used to verify the measurement invariance of Chinese HEMA-R across gender. We chose configural, metric and scalar invariance, and the indices were the changes for CFI and RMSEA (ΔCFI and ΔRMSEA). The measurement invariance was thought to exist if ΔCFI < 0.01 and ΔRMSEA < 0.015 [32].

### 2.2. Results

#### 2.2.1. Confirmatory Factor Analysis

Before performing CFA, we tested the multivariate normality of this data. Mardia’s normalized estimate of multivariate kurtosis was used to assess the multivariate normality distribution [33]. The critical ratio of kurtosis was considered to be acceptable with a value less than 5.00 [34]. In this study, the value of multivariate kurtosis was 160.34 and its critical ratio was 170.90, which indicated that the data didn’t fit the multivariate normality distribution. Thus, we applied robust maximum likelihood (MLR) estimation method due to it being suitable for both complete and incomplete non-normal data.

Then we completed a CFA in order to examine whether the two-factor structure exists in the HEMA-R. The results indicated that all of the model fit indices met the criteria, *χ^2^* (34) = 147.676, *p* < 0.001, CFI = 0.959, NNFI = 0.946, RMSEA = 0.055, SRMR = 0.034. For the purpose of improving the model, we examined the modification indices. We allowed errors of item 6 (“Seeking enjoyment?”) and item 7 (“Seeking to take it easy?”) to correlate. Then, the CFA was exerted again and the modified model showed a greater fit, *χ^2^* (33) = 98.072, *p* < 0.001, NNFI = 0.968, CFI = 0.977, RMSEA = 0.043, SRMR = 0.027. The factor loadings ranged from 0.72 to 0.87 (*p* < 0.001).

Some studies showed that the two-factor structure did not fit the data well using CFA. Besides, researchers found a three-factor model which contained eudaimonic factor, hedonic pleasure factor and hedonic comfort factor [12,13]. In the present study, we also tested the three-factor model, which also fit the data, *χ^2^* (32) = 136.857, *p* < 0.001, NNFI = 0.947, CFI = 0.962, RMSEA = 0.055, SRMR = 0.033. Significant changes occurred when compared to the modified two-factor model, ∆CFI = 0.015, ∆RMSEA = 0.012. All these demonstrated that the two-factor model showed a better result than the three-factor one. Thus, we selected the modified two-factor model as the final model.

#### 2.2.2. Measurement Invariance Across Gender

Firstly, the configural invariance was adopted to test whether the two-factor structure existed in the Chinese HEMA-R across gender. The results of the configural invariance model (Model 1) showed a great fit to the data, CFI = 0.971, RMSEA = 0.051 (other results in Table 1). All factor loadings reached significant levels (*p* < 0.001). All of the above suggested that the data in both gender groups showed a two-factor model. 

Next, in order to examine the metric invariance model (Model 2), factor loadings between two gender groups were constrained to be equal. The results revealed a good fit, CFI = 0.967, RMSEA = 0.051 (other indices in Table 1). And compared to Model 1, no significant changes were found, ΔCFI = 0.004, ΔRMSEA = 0.000. Thus, the metric invariance is held across two gender groups.

Subsequently, we further constrained intercepts to be equal across different groups to establish the scalar invariance (Model 3). The results revealed that this model had a great fit, CFI = 0.965, RMSEA = 0.050 (other indices in Table 1). Then we compared this model to Model 2 and no significant changes were found, ∆CFI = 0.002 and ΔRMSEA = 0.001. These findings suggested that the invariance of intercepts has remained in two gender groups.

Generally speaking, measurement invariance existed across gender.

#### 2.2.3. Scale Reliability

We first tested Cronbach’s alpha coefficients of HEMA-R and found those of two subscales were satisfactory (0.903 for hedonic motives, and 0.901 for eudaimonic motives). Besides, the McDonald’s omega coefficients were also used and showed satisfactory results (0.905 for hedonic motives and 0.902 for eudaimonic motives). Furthermore, we examined the average variance extracted (AVE) and composite reliability (CR). The AVE of the two subscales was 0.602 and 0.602, which were greater than 0.50 [35]. The CR of them was 0.883 and 0.882 (>0.70) [35]. These indicate that all values of the coefficients were acceptable.

## 3. Study 2

### 3.1. Methods

#### 3.1.1. Participants and Procedure

The participants included 751 undergraduates from one school in China (339 males). In study 2, the mean age of the participants is 19.85 ± 1.07 and ranged from 17 years old to 29 years old, and three participants had no age information. Before participating in the survey, all participants signed a written informed consent. Then, they completed questionnaires through a survey website (www.wjx.cn).

#### 3.1.2. Measurement

To assess social support, the Chinese version of Multi-dimensional Scale of Perceived Social Support (MSPSS) was chosen in this research. It was first developed by Zimet and Dahlem [36], including 12 items. MSPSS is a self-administered measurement tool with three subscales: Family Support, Friends Support and Significant-other Support. Every item was scored on a 7-point Likert scale ranging from 1 (very strongly disagree) to 7 (very strongly agree). Besides, MSPSS has shown great reliability and validity in Chinese samples [37,38]. In the present study, the Cronbach’s alpha coefficient of the MSPSS was 0.92.

To test for addiction to smartphones, we used the Chinese version of Smartphone Addiction Scale-Short Version (SAS-SV). The first version of SAS-SV was developed by Kwon, Kim [39]. The scale included 10 items, each uses a 6-point Likert scale ranging from 1 (strongly disagree) to 6 (strongly agree). It has been validated in the Chinese sample by some researchers [40,41]. The Cronbach’s alpha coefficient of the SAS-SV in our study was 0.92.

We used the Chinese version of the Mental Health Continuum-Short Form (MHC-SF) to test the participants’ well-being. It was adapted from the long form of Mental Health Continuum, which was developed by Keyes [42]. There are 14 items in MHC-SF with three dimensions of well-being: hedonic well-being, social well-being and eudaimonic well-being. Participants were asked about their feelings in the past month and scored them on a 6-point Likert scale ranging from 1 (never) to 6 (every day). The MHC-SF was reliable and has shown fine validity in the Chinese sample [43,44]. In the present study, the Cronbach’s alpha coefficients of hedonic well-being, social well-being and eudaimonic well-being were 0.86, 0.84, and 0.93, respectively.

#### 3.1.3. Data Analysis

First, we conducted a CFA to verify whether the two-factor structure existing in HEMA-R using another Chinese sample. The same indices in Study 1 were adapted to reflect the model fit.

After that, a multi-group CFA was performed in order to assess the measurement invariance across gender.

At last, the relationships among hedonic motives, eudaimonic motives, hedonic well-being, social well-being, eudaimonic well-being, social support and smartphone addiction were examined by Pearson correlations.

### 3.2. Results

#### 3.2.1. Confirmatory Factor Analysis

In Study 1, the multivariate kurtosis was 82.61 and its critical ratio was 73.07, which was no less than 5.00. We then chose MLR in subsequent analysis. In Study 2 we also allowed errors of item 6 and item 7 to correlate, which was the same as Study 1. The results of CFA indicated that all of the model fit indices met the criteria, *χ^2^* (33) = 165.825, *p* < 0.001, CFI = 0.939, NNFI = 0.917, RMSEA = 0.073, SRMR = 0.050. The factor loadings ranged from 0.67 to 0.85 (*p* < 0.001).

#### 3.2.2. Measurement Invariance Across Gender

Firstly, we tested the configural invariance (Model 4) with the aim of examining whether the two-factor structure existed in the Chinese HEMA-R across gender. It was shown that the model has a great fit; CFI = 0.934, RMSEA = 0.055 (other results in Table 2). Besides, all factor loadings reached a significant level (*p* < 0.001). All the above indicated that the two gender groups showed a two-factor model.

After that, we confirmed whether the factor loadings were equivalent across two groups in order to examine the metric invariance model (Model 5). The results suggested an acceptable fit; CFI = 0.929, RMSEA = 0.063 (other results in Table 2). We then compared it to Model 4; ΔCFI = 0.005, ΔRMSEA = 0.003, and found both were less than the critical value. These results indicated that the factor-loading invariance existed across gender.

Finally, we further constrained intercepts to be equal across two groups in order to establish scalar invariance (Model 6). With the results of CFI = 0.924, RMSEA = 0.075 (other results in Table 2), the model was acceptable. There were no significant changes compared to Model 5; ΔCFI = 0.005 and ΔRMSEA = 0.001. These findings suggested that intercepts remained invariant in the two gender groups.

In sum, all the results represent that the measurement invariance of the HEMA-R exists in two gender groups.

#### 3.2.3. Descriptive Analysis and Scale Reliability

Table 3 showed the means and standard deviations of hedonic motives, eudaimonic motives, hedonic well-being, social well-being, eudaimonic well-being, social support and smartphone addiction. The Cronbach’s alpha coefficients of HEMA-R were 0.880 for hedonic motives, and 0.872 for eudaimonic motives. The McDonald’s omega coefficients of them were 0.882 and 0.874, respectively. The AVE of the two subscales was 0.619 and 0.629. The CR of them was 0.890 and 0.894. These indicate that all values of the coefficients were acceptable.

#### 3.2.4. Predictive Validity

To examine the predictive validity of the HEMA-R, Pearson’s correlation was chosen to verify its relations with hedonic well-being, social well-being, eudaimonic well-being, social support and smartphone addiction. The results revealed that both components of HEMA-R were positively correlated with hedonic well-being, social well-being, eudaimonic well-being, social support and smartphone addiction (*p* < 0.01), except that eudaimonic motives were negatively correlated with smartphone addiction (*p* < 0.01) (the specific values are reported in Table 4).

In addition, we tested whether the correlation between the eudaimonic motives and the variables has a significant difference with the correlation between hedonic motives and the variables using the Fisher r-to-Z transformation (Table 4). There were significant differences in correlation coefficients in social well-being (Z = 3.79, *p* < 0.001), eudaimonic well-being (Z = 5.22, *p* < 0.001), and smartphone addiction (Z = −6.08, *p* < 0.001). Specifically, eudaimonic motives had a higher correlation with social well-being and eudaimonic well-being than hedonic motives, but had a lower correlation with hedonic motives. There were no significant differences in correlation coefficients in hedonic well-being (Z = 1.83, *p* > 0.05) and social support (Z = 1.34, *p* > 0.05), suggesting both eudaimonic and hedonic motives had an equal correlation with hedonic well-being and social support.

## 4. Discussion

The purposes of the current study were to verify its construct and predictive validity as well as measurement invariance across gender of the Chinese version of HEMA-R. Our findings suggested that the Chinese version of HEMA-R had acceptable reliability in both hedonic and eudaimonic motives. Besides, the two-factor structure was replicated in two Chinese samples, which is consistent with the previous study by Huta and Ryan [9]. Then, the measurement invariance was held across gender groups in two studies. Finally, the HEMA-R had satisfactory predictive validity with measures of well-being, social support and smartphone addiction. All of these indicated that the HEMA-R is a reliable and valid tool when measuring hedonic and eudaimonic motives of Chinese adults.

In this research, one extension was that we tested the measurement invariance across gender. Multi-group CFA results manifested that configural, metric and scalar invariance existed, which is similar to Asano and Igarashi [19]. All these indicated that the HEMA-R can be utilized in different genders. As far as we know, this is the first time the measurement invariance across gender of HEMA-R in Chinese has been examined.

Furthermore, we tested the associations among hedonic and eudaimonic motives, hedonic well-being, social well-being, eudaimonic well-being, social support and smartphone addiction. First, eudaimonic motives exerted a greater effect on eudaimonic aspects of well-being including psychological well-being and social well-being. This was similar to Zeng and Chen [26]. These may suggest that pursuing eudaimonia promotes better well-being than pursuing hedonia, eudaimonia and hedonia may foster well-being in different ways [14]. Second, hedonic and eudaimonic motives were both positively related to social support, and there was no significant difference between them, which suggests that hedonic and eudaimonic motives play an equally important role in social support. These findings are similar to the study by Asano who found that the components of the HEMA were positively related to social support (Asano et al. 2014). However, they didn’t test whether eudaimonic motives have a greater impact on social support than hedonic motives, which was confirmed in our study.

Finally, we found something intriguing about the associations between the two types of motives and smartphone addiction. Hedonic motives were positively related to smartphone addiction while eudaimonic motives were negatively correlated with it. Chen and Zhang [45] found that perceived enjoyment and pastimes positively affected smartphone addiction. In this way, individuals with high hedonic motives tend to choose things that bring enjoyment, such as smartphones, so they are more likely to get addicted to mobile phones. While Cevik and Cigerci [46] revealed a significant negative correlation between smartphone addiction and the meaning of life, which may indicate that individuals with high eudaimonic motives prefer to pursue more meaningful things than spending time on smartphones. Thus, hedonic motives and eudaimonic motives showed different correlation patterns with smartphone addiction.

All in all, the present research provided some new insights into hedonic and eudaimonic motives literature. First, the Chinese version of HEMA-R had satisfactory internal consistency and structure validity, indicating that investigators can utilize the HEMA-R with the aim of measuring how people pursue well-being in a Chinese sample. Second, the configural, metric and scalar invariance in different gender groups were held in this scale, meaning there is no need to consider the influence of gender when using it. Third, the Chinese HEMA-R had acceptable predictive validity with measures of hedonic well-being, eudaimonic well-being and social support which reinforces previous research. Furthermore, the results of social support and smartphone addiction broadened the research approaches of hedonic and eudaimonic motives. Fourth, we conducted two studies, both proved HEMA’s CFA fit well, which to some extent verified the repeatability of the results and the stability of the scale. Furthermore, this instrument could also be useful in examining associations with the other two facets of happiness; fluctuating and durable-authentic happiness [47].

Even though this research assessed the satisfactory results, there some limitations exist that need our attention. First, this study used a Chinese sample whose age was between 17 years to 29 years, so whether the findings can be generalized to other ages or cultural populations needs to be tested in the future. Second, we only examined the gender invariance, other aspects which may influence the results should be tested in the future, such as age. At last, the cross-sectional data couldn’t make sure that the findings still hold when data was collected from different times, so it is meaningful to test the longitudinal data.

## 5. Conclusions

The findings of these results support that the Chinese adaptation of HEMA-R is valid and reliable. It can be utilized in Chinese adults to assess their hedonic and eudaimonic motives regardless of their gender, which is similar to previous studies from other countries. Furthermore, this research helps to fill the gap in knowledge of the measurement of hedonic and eudaimonic motives in cultures around the world.

## Figures and Tables

**Table 1:** Measurement invariance across gender.

Invariance Model	*χ^2^*	*df*	CFI	NNFI	RMSEA	SRMR	Comparison	ΔCFI	ΔRMSEA
Model 1	155.752	64	0.971	0.959	0.051	0.032			
Model 2	177.247	74	0.967	0.960	0.051	0.044	2verses1	0.004	0.000
Model 3	192.781	82	0.965	0.962	0.050	0.043	3verses2	0.002	0.001

**Table 2:** Measurement invariance across gender.

Invariance Model	*χ^2^*	*df*	CFI	NNFI	RMSEA	SRMR	Comparison	ΔCFI	ΔRMSEA
Model 4	212.347	64	0.934	0.907	0.079	0.055			
Model 5	232.455	74	0.929	0.914	0.076	0.063	5verses4	0.005	0.003
Model 6	253.039	82	0.924	0.916	0.075	0.068	6verses5	0.005	0.001

**Table 3:** Mean, SD and alpha of the variables.

Variables	M	SD	Alpha
HM	26.94	4.96	0.88
EM	26.30	4.86	0.87
HWB	12.97	2.76	0.86
SOWB	20.47	4.88	0.84
EWB	25.63	5.94	0.93
WB	59.07	12.38	0.94
SS	44.46	7.71	0.92
SA	35.08	9.80	0.92

M = mean; SD = Standard Deviation; Alpha = Cronbach’s alpha; HM = hedonic motives; EM = eudaimonic motives; HWB = hedonic well-being; SOWB = social well-being; EWB = eudaimonic well-being; WB = well-being; SS = social support; SA = smartphone addiction.

**Table 4:** Correlation among variables and the difference of the correlation coefficients.

Variables	HWB	SOWB	EWB	SS	SA
eudaimonic motives	0.357 **	0.422 **	0.482 **	0.355 **	−0.164 **
hedonic motives	0.272 **	0.249 **	0.250 **	0.293 **	0.148 **
Z	1.83	3.79 ***	5.22 ***	1.34	−6.08 ***

HWB = hedonic well-being; SOWB = social well-being; EWB = eudaimonic well-being; WB = well-being; SS = social support; SA = smartphone addiction. ** *p* < 0.01. *** *p* < 0.001.

## Data Availability

The data presented in this study are available on request from the corresponding author. The data are not publicly available due to the privacy of the participants.
